# Positional Differences in Physique, Physical Strength, and Lower Extremity Stability in Korean Male Elite High School Basketball Athletes

**DOI:** 10.3390/ijerph19063416

**Published:** 2022-03-14

**Authors:** Ju-Yong Bae

**Affiliations:** Department of Physical Education, Dong-A University, Busan 49315, Korea; kosa99@dau.ac.kr

**Keywords:** athletic training, youth athletes, basketball player, physical fitness, Y-Balance Test

## Abstract

No studies have measured the physical strength and lower extremity stability of elite male high school basketball players. This study aimed to measure the physique, physical strength, and lower extremity stability of such athletes in Korea and analyze the differences according to their play positions. Overall, 204 male elite basketball players participated and were classified as guard (*n* = 97), forward (*n* = 69), and center (*n* = 38) according to their main playing position. All sub-variables of physique were significantly higher in the forward and center groups than in the guard group, and were significantly higher in the center group than in the forward group. Strength was significantly higher in the forward and center groups than in the guard group. Agility and speed were significantly faster in the guard group than in the forward and center groups. Y-balance analysis showed that the composite score of both feet tended to be higher in the order of center, forward, and guard, and it was significantly higher in the guard group than in the center group. These results could be used as basic data for selecting players, determining positions, and setting specific training goals for players of each position to improve physical strength and prevent injuries.

## 1. Introduction

Developing a potential athlete by discovering and improving the talents of youth athletes is a very important concern for coaches of all sports, and is recognized as an essential quality of a good leader in the sports fields [1]. To become a competent athlete in the sports field, multifactorial qualities, such as anthropometric, physiological, technical, tactical, psychological, and environmental aspects, are required, and the importance of qualities differs slightly depending on the characteristics of each sport [2]. In the case of basketball, height [3], physical fitness [4], and skill [5] may be useful predictors in discovering young players. Therefore, the Korean Basketball League (KBL) has been conducting the “Youth Player Development Project” every year since 2019 to discover and support elite youth basketball players. This project selects excellent players by measuring the physique and physical strength of youth basketball players, and then supports them to become excellent professional players by teaching them basketball skills [6].

Basketball is a team-based sport that requires collaboration between team players with distinct roles and abilities. Play positions are determined based on the athlete’s physique, fitness, and skills, and athletes perform necessary roles in specific positions on the basketball court [7]. Traditionally, three (guard, forward, and center) or five positions (center, power forward, small forward, point guard, and shooting guard) are used in a basketball game [8]. Previous studies reported that anthropometric and physiological factors of players differ between the play positions. Centers are taller and heavier than players in other positions [9], and guards have superior running abilities, such as sprinting and shuttle running, compared to other positions [10,11].

However, basketball players are exposed to the risk of injury because of the nature of the sport, in which frequent physical contact occurs in a narrow space. In particular, basketball players frequently perform vertical jumps, landings, and lateral shuffling, compared to players in other sports, so the risk of injury to the lower extremities is higher than that of the upper extremities [12]. The most common types of injuries in basketball players are anterior cruciate ligament tears and ankle sprains [13]. Therefore, efforts to evaluate and improve lower extremity stability are important to predict and prevent injuries in basketball players. A representative method for evaluating lower extremity stability is the Lower Quarter Y-Balance Test (LQ-YBT). LQ-YBT measures lower extremity stability by having an individual stand on one foot on a centrally located platform and extend the other foot as far as possible in the anterior, post-medial, and post-lateral directions [14].

The play positions of most youth basketball players are determined during high school when the physical growth of athletes has stagnated. Therefore, identifying and understanding the proper physique and required physical strength are important for athletes to perform in their position successfully. Although knowing the stability of one’s lower extremities is important to predict and prevent the risk of injury, no studies have measured the physical strength and lower extremity stability of elite male high school basketball players. Hence, studies for selecting new players, discovering potential players, determining positions, setting training goals, and predicting and preventing injuries for male elite high school basketball players are necessary. This study aimed to measure the physique, physical strength, and lower extremity stability of Korean male elite high school basketball athletes registered with the Korea Basketball Association (KBA), and to explore the differences according to the play positions.

## 2. Materials and Methods

### 2.1. Ethics Statements

This study was approved by the Institutional Review Board (IRB) of Dong-A University (IRB number: 2-1040709-AB-N-01-202107-HR-057-02).

### 2.2. Participants and Position Classification

This cohort study included 229 players from 23 teams of Korean male high schools, officially registered with the KBA, that participated in the KBL’s “Youth Player Development Project.” Informed consent was obtained from all participants involved in the project. This study was conducted with the data obtained through this project provided by the KBL.

Play positions were classified into guard (G), forward (F), or center (C), and the coach and manager were asked to write only one position that athletes mainly played during the game. Table 1 shows the general characteristics of players by their position.

### 2.3. Experimental Procedure

All measurements were performed from June 2020 to October 2020. The same three experts visited the gymnasium of each school and used the same measuring equipment. Participants underwent the measurements after performing a sufficient warm-up exercise freely, and measurements were completed in one day in the order of physique, Y-balance, and physical strength. To comply with Korea’s response to COVID-19, participants were asked to wear a medical mask in all procedures except when their physical fitness was measured.

#### 2.3.1. Physique Measurements

The physique measurements were taken in the same way as described in a previous study [6,15], and the specific methods are as follows.

Height was measured using a height meter with 0.1 cm accuracy (HIE-401, Hanil Sporex, Pochen, Korea), and body weight was measured using a digital scale with 0.1 = kg accuracy (HE-17, CAS, Yangju, Korea). Limb length was measured from the sole of the foot to the apex of the forearm using a tape measure with the participant in a comfortable supine position. Standing reach was measured with the participant in a comfortable standing state using the Vertec Vertical Jump Trainer (Sports Imports, Hilliard, OH, USA) with 0.1 cm accuracy.

#### 2.3.2. Y-Balance Test

The Y-Balance Test (YBT), a representative method for measuring lower extremity stability, such as chronic ankle instability and lower extremity mobility [16], and the professional YBT Kit (Functional Movement Systems, Chatham, MA, USA), were used. Participants were asked to extend one leg as far as possible in three directions, anterior, post-medial, and post-lateral, with the other leg supported on a central platform without shoes. The measurement was considered unsuitable when the supported foot fell off the platform, the outstretched foot touched the ground or the measuring device, or the outstretched foot did not return to the starting position. Both feet were measured twice with 0.1 cm accuracy, and a high value was recorded. If both feet failed, remeasurements were performed until success. Composite stability result values (left result and right result) of each foot were calculated and expressed as a composite score for error correction due to leg length; a higher value means better lower extremity stability.

Composite score = [(AT + PM + PL directions)/(3 × Limb length)] × 100

where: AT: anterior, PM: post-medial, PL: post-lateral

#### 2.3.3. Physical Strength Measurements

The physical strength measurements were conducted in the same way as described in a previous study [6,15]. The measurements were performed in the following order: grip strength, back strength, jump test, lane agility, 10-yard sprint, 3/4 court sprint, and 300-yard shuttle run.

Grip strength was measured using a GRIP-D grip dynamometer (Takei, Niigata, Ja-pan) with 0.1 kg accuracy. Participants stood naturally, and the measurement was performed with their arms extended and approximately 10° away from the body. The length of the handle was adjusted to the optimal position according to the length of their hand. After the start signal, grip strength was measured for 3 s while maintaining the starting position.

Back strength was measured using a T.K.K.5402 back strength measuring instrument (Takei, Niigata, Japan) with 0.1 kg accuracy. Participants stood naturally on the dynamometer platform, and held the handle while extending their knees fully straight and bent backwards at approximately 30°. After the start signal, the participants pulled the handle for 3 s without bending their knees while looking straight ahead.

Vertical and running jumps were measured using a Vertec Vertical Jump Trainer (Sports Imports) with 0.1 cm accuracy. The vertical jump was measured under the measuring device, and the running jump was measured by running freely at a distance of 5 m from the device. Participants were asked to touch the wing of the Vertec as high as possible, and the highest touched point was expressed as a vertical jump total and a running jump total. The values obtained by subtracting the standing reach from each record were presented as the vertical jump and running jump records.

Muscle strength and jump tests were conducted twice in total, with a 1 min rest between each trial, and the best record was used in the analysis.

Lane agility, 10-yard sprint, 3/4 court sprint, and 300-yard shuttle run were measured using a stopwatch (SEIKO, Tokyo, Japan) with 0.01 s accuracy. The lane agility test is a specialized test method to measure the agility of basketball players. To measure the lane agility, marker cones are placed at the vertices of a rectangular shape with a width of 4.87 m and a length of 5.79 m. Thereafter, the participants stand on the lower right starting line. At the start signal, the participants sprint with the cone on the top right, then move to the side shuffle with the cone on the top left, backstep with the cone on the bottom left, then go to the side shuffle with the cone on the bottom right (starting line). Then, the time to return to the starting point is measured around the cone in the reverse order (lower left, upper left, upper right, lower right).

For the 10-yard sprint, the time to run a straight-line distance of 9.14 m was measured, and for the 3/4 court sprint, the time it took to run a straight-line distance of 22.86 m was measured.

The 300-yard shuttle run is a test of measuring the anaerobic endurance of athlete. To measure the 300-yard shuttle run, marker cones are placed at the starting line and 25 yards ahead of the starting line. At the start signal, participants start from the starting line, and sprint 25 yards 12 times without stopping (300 yards total), and the arrival times are measured.

Lane agility, 10-yard sprint, 3/4 court sprint, and 300-yard shuttle run were measured twice in total, with 5 min rest between each trial, and the best record was used in the analysis.

### 2.4. Statistical Analysis

Data are reported as the mean ± standard error. One-way analysis of variance followed by the Tukey honest significant difference post hoc test was conducted to compare differences between the groups. The homogeneity of variance of all data was confirmed using the Levene test. All statistical analyses were performed using SPSS, version 22.0 (IBM Corp., Armonk, NY, USA), and statistical significance was set at 0.05.

## 3. Results

### 3.1. Differences in Physique According to the Play Position of Male Elite High School Basketball Athletes

The physique of male elite high school basketball athletes by position is shown in Table 2. Differences in physique according to the play positions were observed in all sub-elements of physique. Height, weight, limb length, wingspan, and standing reach were higher in the forward and center groups than in the guard group (all, *p* < 0.001), and significantly higher in the center group than the forward group (height, weight, wingspan, and standing reach, *p* < 0.001; limb length, *p* < 0.01).

### 3.2. Differences in Physical Strength According the Play Position of Male Elite High School Basketball Athletes

The physical strength of male elite high school basketball athletes by position is shown in Table 3. Grip strength and back strength were significantly higher in the forward and center groups than in the guard group (both, *p* < 0.001), and grip strength was significantly higher in the center group than in the forward group (*p* < 0.05). However, grip strength and back strength normalized by body weight were not significantly different between the groups. Push-up number was significantly higher in the guard and forward groups than in the center group (*p* < 0.001). Vertical jump total and running jump total were significantly lower in the guard group than in the forward and center groups (both, *p* < 0.001), and significantly higher in the center group than in the forward group (vertical jump total, *p* < 0.001; running jump total, *p* < 0.01). However, vertical jump and running jump (jump total minus standing reach) were not significantly different between the positions. The lane agility time was significantly slower in the center group than in the guard (*p* < 0.001) and forward groups (*p* < 0.05), and the 3/4 court sprint time was faster in the guard group than in the center group (*p* < 0.01). The 300-yard shuttle run time was significantly faster in the guard group than in the center group (*p* < 0.05).

### 3.3. Differences in Lower Extremity Stability According to the Play Position

The lower extremity stability of male elite high school basketball athletes by position is shown in Table 4. The anterior right time was significantly longer in the forward group than in the guard group (*p* < 0.05). Additionally, post-medial left, post-medial right, post-lateral left, and post-lateral right direction times were significantly longer in the forward (post-lateral left, post-lateral right, *p* < 0.05; post-medial left, post-medial right, *p* < 0.01) and center groups (post-lateral left, *p* < 0.05; post-lateral right, *p* < 0.01; post-medial left, post-medial right, *p* < 0.001) than in the guard group. The composite score of both feet was higher in the guard group than in the center group (composite right, *p* < 0.01; composite left, *p* < 0.001), and there was a tendency for it to increase in the order of center, forward, and guard. Regarding the difference in the reach of both feet (asymmetry), we found that asymmetry of the post-medial direction was higher in the center group than in the forward group (*p* < 0.05). Asymmetry of the anterior direction was not statistically different between the groups, but there was a tendency for it to increase in the order of center, forward, and guard.

## 4. Discussion

The main findings of this study are as follows. All sub-variables of physique were significantly higher in the forward and center groups than in the guard group, and were significantly higher in the center group than in the forward group. Strength was significantly higher in the forward and center groups than in the guard group. Agility and speed were significantly faster in the guard group than in the forward and center groups. Y-balance analysis showed that the composite score of both feet tended to be higher in the order of center, forward, and guard, and it was significantly higher in the guard group than in the center group.

In a basketball game, performance is determined by a player’s physical, technical, and tactical factors, and a player’s physical characteristics, including physique and physical strength, are the top factors considered by coaches when determining team tactics and positioning players [8,17]. Among the physical factors, physique has the highest priority in the evaluation and selection of players, and height and weight are very important when determining a player’s play position [18,19]. Wingspan and standing reach also affect performance, and these factors help predict whether an athlete will reach the highest level [19]. A comparative analysis of the physiques of 3610 National Basketball Association reserve players who were and were not drafted found that drafted players were taller and had longer wingspans than undrafted players in all five play positions [20]. Therefore, physical superiority acts as an advantage even among players in the same positions. In the present study, all sub-elements of physique were significantly higher in the order of guard, forward, and center, and this result is consistent with those of prior studies. Most previous studies reported that, in terms of physique, the forward has a physique advantage compared to the guard, and the center has a physique advantage compared to the forward [20,21,22]. The height, weight, and length of the arms of the center are used as an advantage in performing the role of the center position, such as rebounding and screening play under the goal [23]. However, the guard requires fast movement, speed, and agility for quick attacks and to return to defense, rather than the advantage of height and weight [20,24].

Basketball is one of the fastest team sports and is characterized by excellent movements, such as sprints, turns, dunks, rebounds, and blocking [25]. Because basketball is characterized by high-intensity exercise at a high frequency, such as in the case of a fast attack for a short period [26], the ability to continuously perform intermittent high-intensity movements and to recover quickly is important for basketball players [27]. In particular, the game speed becomes much faster after the attack time is changed to 24 s, which places high physical demands on players on both defense and attack [28]. Basketball players perform about 1000 high-intensity moves per game, moving 4–6 km [29] and performing 45 sprint moves per minute including two jumps [30]. Therefore, physical strength is an essential factor, along with physique, to become a successful athlete. In this study, the forward and center players showed higher strength and muscular endurance than the guard players, but the guard players were faster than the center players in terms of agility, reflexes, and anaerobic endurance during running. However, there was no significant difference in vertical jump and running jump according to the play position. A previous study reported that muscle strength was higher in the forward and center positions than in the guard position, and the guard position showed higher agility and anaerobic endurance than the other positions [31]. However, the guard position showed better sprint and agility than the center position [21]; this advantage means that guards can frequently perform repetitive high-intensity activities, such as fast attacks and quick returns to defense [23]. In fact, the guard spends a lot of time in possession of the ball during the game compared to other positions and performs more activities at all intensities than forwards and centers, so a guard position requires a high level of agility and the ability to perform high-intensity interval movements [32]. The roles of basketball positions are clearly defined, and the standards of physique and physical strength suitable for successful performance are standardized to some extent. The present study’s results are similar to those of previous studies because the male elite high school basketball players participating in this study already had their play position determined based on their physique and physical strength. Nevertheless, this study has value in that it provides representative data of most Korean male elite high school basketball players.

Basketball players have a high risk of injury to the lower extremities, such as the knees and ankles [33], because the movements performed by players in basketball games are based on a combination of movement in two planes: horizontal movement (sprinting and changing direction) and vertical movement (jump shooting, rebounding, and blocking) [26]. Deficiency in dynamic neuromuscular control of the lower extremities is associated with an increased risk of non-contact injury, and a decrease in control ability is observed even after lower extremity injury occurs [34,35]. Therefore, evaluation of dynamic neuromuscular control is an important process in identifying athletes at risk for injury and preventing injury. The Star Excursion Balance Test (SEBT) and LQ-YBT are widely used in sports fields to evaluate the dynamic neuromuscular control ability of the lower extremities. In a study that used the SEBT to evaluate preseason dynamic balance in high school basketball players, players (both men and women) with an asymmetric forward reaching score of 4 cm or greater were more than twice as likely to experience lower extremity injuries, and female players with lower overall scores had a 6.5 times higher risk of injury than those without [36]. Another study reported that a difference in the reach of both feet of 2 cm or more could predict an ankle sprain [37]. However, some studies reported that the YBT score alone cannot predict injury, which is somewhat controversial [16,38]. Because most epidemiologic investigational studies on injury include injuries caused by contact mechanisms and non-contact injuries caused by a decrease in neuromuscular control ability, the direct relationship between lower extremity stability and injury occurrence remains unclear [39]. Although controversial, a recent review analyzing the reliability of the YBT as an injury prediction tool revealed that the dynamic neuromuscular control ability clearly represents lower extremity stability [40]. In the current study, the composite score of both feet was significantly higher in the guard position than in the center position, and the tendency of higher lower extremities stability was confirmed in the order of guard, forward, and center. In contrast to the composite score, the difference in the forward reach of both feet that predicted injury [36,37] for the guard position was more than 4 cm, and there was a tendency for the difference to be higher in the order of guard, forward, and center. These results indicate that guards have a high risk of injury due to the role of their position, but they also have excellent lower extremity stability and mobility. Therefore, to improve performance and reduce the risk of injury, guards need to make efforts to reduce the asymmetry of both feet.

This study has several limitations. First, the play position of the participants in this study was determined by the coach. There were players who played two positions because they were still growing and/or because of team tactics, but mainly the coaches selected the athletes’ positions. Second, an epidemiological investigation of the athletes’ history of injury and current physical conditions was not conducted. Because it is unclear whether the results were the athletes’ best records, the relationship between injury and lower extremity stability cannot be discussed. However, the data used were obtained from participants who underwent precise measurements and were encouraged to do their best during the measurements. Finally, several statistical tests were performed in this study, raising possible concerns about Type 1 errors; as such, the findings emanating from these comparisons are deemed exploratory and should be considered with caution. Despite these limitations, this study provides novel information on Korean male elite high school basketball players.

## 5. Conclusions

This study suggests differences exist in physique, physical strength, and lower extremity stability according to the position of Korean male elite high school basketball players. This study’s results could be used as basic data for selecting potential athletes, determining positions, and setting specific training goals for players of each position to improve physical strength and prevent injuries.

## Figures and Tables

**Table 1:** General characteristics of players by their position.

	G (*n* = 97)	F (*n* = 69)	C (*n* = 38)	Total (*n* = 204)
Age (Year)	17.03 ± 0.80	17.22 ± 0.82	17.37 ± 0.91	17.16 ± 0.08
Grade	1.80 ± 0.86	1.91 ± 0.82	1.89 ± 0.76	1.86 ± 0.83

Data presented as mean ± SD. G: guard, F: forward, C: center.

**Table 2:** Differences in physique according to the play position.

	G (*n* = 97)	F (*n* = 69)	C (*n* = 38)	Total (*n* = 204)	SS	df	MS	*F*	*p*
Height (cm)	176.81 ±4.62	187.27 ±4.93 ***	195.24 ±3.73 ***###	183.78 ±8.53	10,537.208	2	5268.604	250.912	0.000
Weight (kg)	70.07 ±7.72	80.55 ±7.98 ***	90.29 ±8.63 ***###	77.38 ±11.10	12,206.793	2	6103.397	95.862	0.000
Limb length (cm)	96.03 ±10.03	103.91 ±3.96 ***	109.45 ±3.49 ***##	101.20 ±9.12	5684.377	2	2842.188	51.072	0.000
Wingspan (cm)	181.68 ±6.42	192.46 ±6.31 ***	202.12 ±5.60 ***###	189.14 ±10.02	12,565.768	2	6282.884	161.380	0.000
Standing reach (cm)	228.20 ±7.15	241.34 ±7.89 ***	252.43 ±6.49 ***###	237.16 ±11.86	17,851.329	2	8925.664	167.635	0.000

Data presented as mean ± SD. G: guard, F: forward, C: center, ***; vs. guard (*p* < 0.001), ##; vs. forward (*p* < 0.01), ###; vs. forward (*p* < 0.001).

**Table 3:** Differences in physical strength according to the play position.

	G (*n* = 97)	F (*n* = 69)	C (*n* = 38)	Total (*n* = 204)	SS	df	MS	*F*	*p*
Grip strength (kg)	40.87 ±5.76	47.26 ±6.40 ***	50.42 ±5.45 ***#	44.81 ±7.08	3119.719	2	1559.859	44.386	0.000
Grip strength (kg/BW)	0.59 ±0.76	0.59 ±0.69	0.56 ±0.66	0.58 ±0.72	0.020	2	0.010	1.954	0.144
Back strength (kg)	132.49 ±22.99	150.72 ±27.20 ***	156.79 ±20.78 ***	143.18 ±26.17	22,061.979	2	11,030.989	18.948	0.000
Back strength (kg/BW)	3.25 ±0.40	3.20 ±0.45	3.13 ±0.41	3.21 ±0.42	0.404	2	0.202	1.135	0.324
Push-up (n)	38.68 ±13.16	38.03 ±12.22	28.05 ±10.98 ***###	36.48 ±13.05	3333.992	2	1666.996	10.726	0.000
Vertical jump total (cm)	288.57 ±9.08	303.69 ±9.18 ***	313.51 ±8.57 ***###	298.33 ±13.38	19,969.677	2	9984.839	122.610	0.000
Vertical jump (cm)	60.37 ±6.59	62.35 ±8.89	61.08 ±7.82	61.17 ±7.30	158.204	2	79.102	1.491	0.228
Running jump total (cm)	306.71 ±10.61	321.19 ±9.93 ***	329.34 ±10.67 ***###	315.82 ±13.81	16,989.510	2	8494.755	78.571	0.000
Running jump (cm)	78.51 ±8.55	79.88 ±9.29	76.91 ±9.73	78.68 ±9.05	221.262	2	110.631	1.357	0.260
Lane agility (s)	11.77 ±0.62	11.96 ±1.46	12.56 ±0.73 ***#	11.98 ±1.04	17.104	2	8.552	8.511	0.000
10 Yard sprint (s)	1.71 ±0.16	1.73 ±0.16	1.75 ±0.16	1.72 ±0.16	0.070	2	0.035	1.342	0.264
3/4 court sprint (s)	3.56 ±0.18	3.63 ±0.17	3.68 ±0.21 **	3.61 ±0.19	0.444	2	0.222	6.572	0.002
300 Yard shuttle run (s)	55.28 ±6.28	56.82 ±8.60	58.86 ±2.72 *	56.47 ±6.82	361.966	2	180.983	4.004	0.020

Data presented as mean ± SD. G: guard, F: forward, C: center, BW: body weight, *; vs. guard (*p* < 0.05), **; vs. guard (*p* < 0.01), ***; vs. guard (*p* < 0.001), #; vs. forward (*p* < 0.05), ###; vs. forward (*p* < 0.001).

**Table 4:** Differences in lower extremity stability according to the play position.

	G (*n* = 97)	F (*n* = 69)	C (*n* = 38)	Total (*n* = 204)	SS	df	MS	*F*	*p*
Anterior L (cm)	56.35 ±6.52	58.67 ±5.71	58.76 ±6.76	57.58 ±6.39	281.299	2	140.650	3.535	0.031
Anterior R (cm)	55.99 ±7.27	58.70 ±6.57 *	57.61 ±6.79	57.21 ±7.02	302.676	2	151.338	3.131	0.046
PostMedial L (cm)	105.54 ±8.90	109.65 ±7.73 **	112.18 ±9.99 ***	108.17 ±9.10	1436.847	2	718.423	9.400	0.000
PostMedial R (cm)	105.65 ±8.39	109.83 ±7.09 **	113.34 ±8.86 ***	108.50 ±8.56	1800.447	2	900.223	13.846	0.000
PostLateral L (cm)	103.58 ±10.80	108.00 ±7.61 *	108.68 ±12.08 *	106.02 ±10.33	1118.997	2	559.498	5.477	0.005
PostLateral R (cm)	104.76 ±9.74	108.87 ±8.53 *	111.68 ±11.63 **	107.44 ±10.07	1520.711	2	760.356	8.016	0.000
Composite L	91.49 ±9.00	88.73 ±5.82	85.30 ±7.75 ***	89.41 ±8.12	1094.182	2	547.091	8.944	0.000
Composite R	91.82 ±8.92	89.07 ±5.90	86.20 ±7.54 **	89.84 ±8.01	924.868	2	462.434	7.674	0.001
Absolute difference Between L and R (cm)									
Anterior	4.15 ±2.82	3.91 ±3.98	3.58 ±2.49	3.97 ±3.20	9.338	2	4.669	0.453	0.636
PostMedial	5.02 ±4.24	4.14 ±3.44	6.63 ±5.20 #	5.02 ±4.26	151.526	2	75.763	4.312	0.015
PostLateral	5.64 ±4.82	6.23 ±4.80	7.21 ±5.26	6.13 ±4.91	68.450	2	34.225	1.428	0.242
Composite	3.20 ±2.49	3.03 ±2.10	3.79 ±3.25	3.25 ±2.53	14.560	2	7.280	1.140	0.322

Data presented as mean ± SD. G: guard, F: forward, C: center, L: left, R: right, *; vs. guard (*p* < 0.05), **; vs. guard (*p* < 0.01), ***; vs. guard (*p* < 0.001), #; vs. forward (*p* < 0.05).

## Data Availability

Data generated and analyzed during this study are included in this article. Additional data are available from the corresponding author on request.
